# Identification of long non-coding RNA *ZFAS1* as a novel biomarker for diagnosis of HCC

**DOI:** 10.1042/BSR20171359

**Published:** 2018-07-18

**Authors:** Ping Luo, Chunzi Liang, Xianwei Zhang, Xuefang Liu, Yingchao Wang, Mengmeng Wu, Xiaobo Feng, Jiancheng Tu

**Affiliations:** 1 Department of Clinical Laboratory Medicine and Center for Gene Diagnosis, Zhongnan Hospital of Wuhan University, Wuhan, China; 2 Department of Pain Management, Zhongnan Hospital of Wuhan University, Wuhan, China; 3 Division of Kidney Disease, Zhongnan Hospital of Wuhan University, Wuhan, China

**Keywords:** Biomarker, Hepatocellular carcinoma, Long non-coding RNAs, ZFAS1

## Abstract

Hepatocellular carcinoma (HCC) is the third major cause of cancer-related deaths. Abundant research show that long non-coding RNAs (lncRNAs) play critical roles in the initiation and progression of HCC and may serve as diagnostic markers for HCC. In the present study, six lncRNAs were chosen as candidate genes on the basis of previous literature to evaluate their diagnostic value on HCC by qRT-PCR. Experiment was first carried out in 22 pairs of tissues from HCC and then those were differently expressed in tissues were further tested in plasma from 20 HCC patients and 20 control cases. At last, *ZFAS1* was chosen to be further analyzed in another 214 plasma samples including 79 control cases, 75 hepatitis B and cirrhosis patients, and 60 HCC patients. The levels of plasma *ZFAS1* in HCC were significantly higher than those in healthy controls (*P*<0.001), and in patients with cirrhosis and hepatitis B (*P*<0.001), and was positively associated with serum α-fetoprotein (AFP). Meanwhile, the area under the receiver operating characteristic curve (AUC) of *ZFAS1* was 0.801 to diagnose HCC from healthy controls, while AFP was 0.798 and the combined AUC of *ZFAS1* and AFP was 0.891 (95% CI: 0.829–0.953), slightly higher than *ZFAS1* alone. In conclusion, our results indicated that *ZFAS1* could serve as a biomarker for diagnosing HCC.

## Introduction

Hepatocellular carcinoma (HCC) is the third major cause of cancer-related deaths and caused approximately over 700000 deaths worldwide each year [1–3]. It is reported that HCC in China alone accounts for >50% of the cases worldwide owing to the prevalence of hepatitis B virus (HBV)-induced hepatitis [4]. Despite the improvement in technology, most HCC patients continue to be diagnosed at advanced stage and the 5-year survival rate is still below 20% [5]. Epidemiologic evidence indicates that during the next decades the burden caused by HCC will still significantly increase [6]. At present, plasma α-fetoprotein (AFP) has been widely used in clinical practice, however, the utility of AFP is limited due to its low sensitivity and specificity [7]. Therefore, it is urgently needed to identify new tumor markers with high sensitivity and specificity to detect HCC independently or as a supplement of AFP.

Long non-coding RNAs (lncRNAs) are a group of RNAs longer than 200 nts in length which have no protein-coding potential [8]. LncRNAs can regulate gene expression at transcriptional, post-transcriptional, and epigenetic levels, and play critical roles in multiple biological processes across every branch of life such as cell growth, differentiation, proliferation, survival, and migration [9]. Notably, emerging evidences have pointed out that dysregulation of lncRNAs can contribute to the progress of various human diseases, especially cancer [10]. Abundant recent research suggests that lncRNAs play critical roles in the initiation and progression of HCC. For instance, up-regulation of lncRNA *LOC90784* can promote cell invasion and proliferation and is associated with poor clinical features in HCC [11]. Furthermore, it is reported that plasma DANCR showed an increased discriminatory power for differentiating HCC patients from non-HCC patients compared with AFP [12]. Therefore, we highly hold the view that lncRNAs can be potential diagnostic markers for HCC.

Clinical biomarkers should be easily accessible and non-invasive. Circulating RNA in plasma or serum has attracted wide attention for its non-invasive feature [13]. Increasing number of studies has demonstrated that circulating lncRNAs have dysregulated in plasma or serum, indicating their high potential as powerful and non-invasive tumor markers [14,15]. For example, *H19* was found to be significantly up-regulated in plasma of gastric cancer patients, and could be used to discriminate gastric cancer patients from healthy controls [16]. LncRNA *POU3F3* could serve as a potential biomarker to diagnose esophageal squamous cell carcinoma [17]. In breast cancer patients, *GAS5* can be used as a potential biomarker to assess the surgical effects [18]. However, few studies investigated circulating lncRNA for early detection of HCC patients.

Here we selected six candidate cancer-related lncRNAs (*ZFAS1* [19], *PRNCR1* [20], *GAS5* [21], *TUG1* [22], *linc-ROR* [23], and *H19* [24]) through articles that were previously reported to be dysregulated in cancer. The aim of our study was to validate whether the lncRNAs we selected were detectable and altered in HCC patients, then to find out whether it is a suitable biomarker to diagnose HCC.

## Materials and methods

### Sample collection

Twenty-two paired fresh tumor tissues and adjacent normal tissues were obtained from HCC patients who underwent surgical resection in Zhongnan Hospital of Wuhan University between January and July 2016. All the tissues were immediately stored at −80°C until RNA extraction. Blood samples from 80 HCC patients (71 males and 9 females, mean age: 58 ± 9), 75 hepatitis B and cirrhosis (45 males and 30 females, mean age: 56 ± 8), and 99 healthy controls (70 males and 29 females, mean age: 54 ± 10) were collected from Zhongnan Hospital of Wuhan University between 2016 and 2017. All subjects were included according to the medical or pathology reports. Patients who accepted any chemotherapy or radiotherapy were not included in our study and all the included healthy controls were free of any liver disease. Plasma samples in EDTA tubes were collected within 12 h from drawn blood. Blood samples were firstly centrifuged at 2000 ***g*** for 5 min at 4°C, then the supernatants were transferred into microcentrifuge tubes and centrifuged at 12000 ***g*** for 10 min at 4°C twice to remove cellular nucleic acids completely.

### Ethical approval

All subjects included in the study signed informed consents. The study was approved by the Ethics Committee of Zhongnan Hospital of Wuhan University (ethical approval number 2013059) and was done according to the Declaration of Helsinki.

### RNA isolation and cDNA synthesis

Trizol reagent (Invitrogen, U.S.A.) was used to extract the RNA of tissues and plasma total RNA was isolated using blood total RNA isolation kit (RP4001, BioTeke, Beijing, China) from 300 μl plasma according to the manufacturer’s instructions. The concentration of RNA was measured by Nanodrop 2000 spectrophotometer (Thermo, CA, U.S.A.). The reverse-transcription reaction was performed using PrimeScript II cDNA synthesis kit with gDNA Eraser (Takara, Dalian, China). The reaction conditions for cDNA synthesis were as follows: 42°C for 2 min to remove the contaminated DNA, and then 37°C for 15 min, 85°C for 5 s.

### Real-time PCR analysis

Quantitative real-time PCR assay was performed on the Bio-Rad CFX96 (Bio-Rad Laboratories, Inc., Hercules, CA, U.S.A.) using SYBR-green I Premix EXTaq following the instructions for users. The reaction protocol was as follows: 95°C for 5 min, followed by 40 cycles of 95°C for 30 s, 63.7°C for 30 s, and 72°C for 30 s. All the primers of lncRNAs used in the present paper were listed in Table 1 and *18S* was used as the endogenous control. Samples were analyzed in duplicate with no-template controls done at the same time.

**Table 1:** Sequences of primers used in the present paper

Name		Sequences
*ZFAS1*	Sense	5′-ACGTGCAGACATCTACAACCT-3′
	Antisense	5′-TACTTCCAACACCCGCAT-3′
*PRNCR1*	Sense	5′-CAGCAGCCGATATGATGGTATT-3′
	Antisense	5′-GGTTCTGTTGTCTGGTGATGG-3′
*GAS5*	Sense	5′-GTGTGGCTCTGGATAGCAC-3′
	Antisense	5′-ACCCAAGCAAGTCATCCATG-3′
*TUG1*	Sense	5′-ACAACGACTGAGCAAGCACTA -3′
	Antisense	5′-GGAGGCACAGGACATAATTCACT-3′
*Linc-ROR*	Sense	5′-CATCCGGCGCTCAGCT-3′
	Antisense	5′-TCATGATGGCTGTATGTGCCA-3′
*H19*	Sense	5′-GGGTCTGTTTCTTTACTTCCTCCAC-3′
	Antisense	5′-GATGTTGGGCTGATGAGGTCTGG-3′
*18S*	Sense	5′-CAGCCACCCGAGATTGAGCA-3′
	Antisense	5′-TAGTAGCGACGGGCGGTGTG-3′

### Statistical analysis

All statistical analyses were performed using the SPSS version 17.0 (SPSS, Inc. Chicago, IL, U.S.A.) and GraphPad Prism 5.0 (GraphPad Software, La Jolla, CA, U.S.A.). Shapiro–Wilk test was used to test the normality of distribution for each dataset. Student’s *t* test was operated to analyze normally distributed data, while non-normally distributed variables were tested by Kruskal–Wallis variance analysis. Receiver operating characteristic (ROC) curves were performed to evaluate the feasibility of using plasma *ZFAS1* as a diagnostic maker. All statistical tests were two-sided and *P*<0.05 was considered statistically significant.

## Results

### Selection of HCC-related lncRNAs

**Figure 1:**
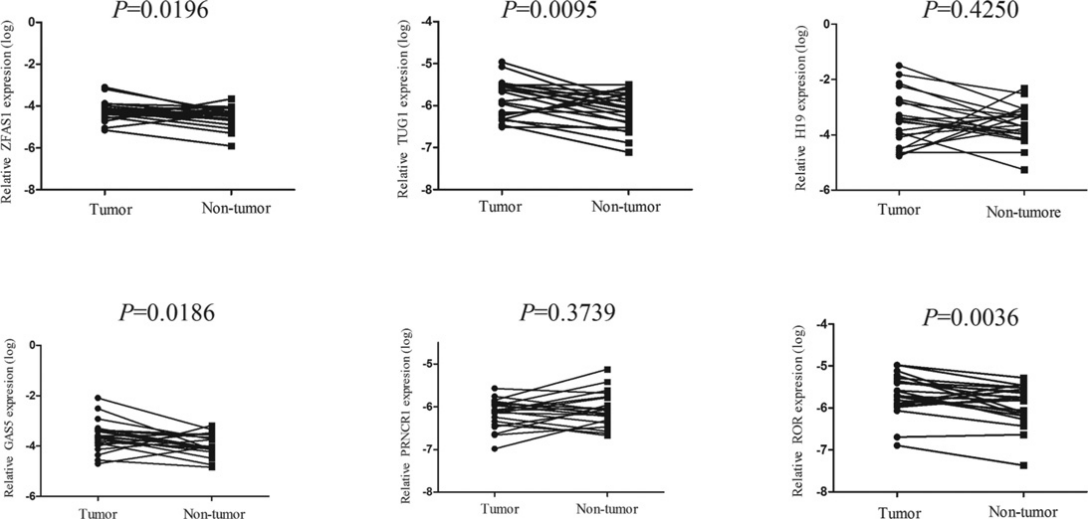
*ZFAS1, TUG1, GAS5*, and *linc-ROR* were selected as the candidate lncRNAs in HCC tissue Increased levels of *ZFAS1, TUG1, GAS5*, and *linc-ROR* were confirmed by qRT-PCR. All data were analyzed using Student’s *t* test.

**Figure 2:**
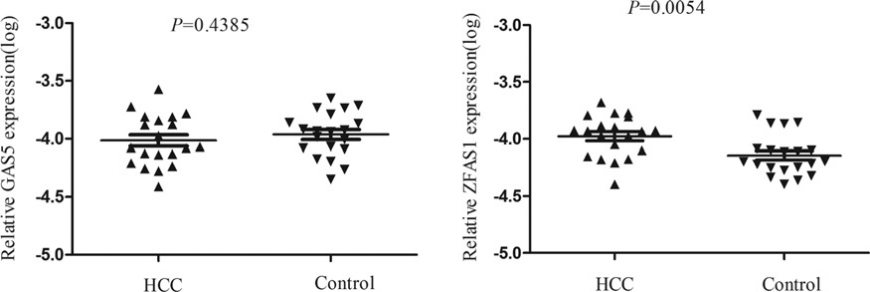
*ZFAS1* were selected as HCC-related biomarkers in plasma *GAS5* showed no significant changes between plasma of HCC patients and controls, while *ZFAS1* was significantly up-regulated in plasma of HCCs. The data were analyzed using Student’s *t* test.

Through searching the published literatures, six lncRNAs (*ZFAS1, PRNCR1, GAS5, TUG1, linc-ROR, H19*) which have been reported to be abnormally expressed in cancer were included in the present study. RT-qPCR was conducted to validate the expression of the six lncRNAs in 22 pairs of HCC and normal tissues. The detailed characteristics of the 22 patients are summarized in Supplementary Table S1. Results showed that *ZFAS1, TUG1, GAS5*, and *linc-ROR* were up-regulated in HCC tissues compared with paired normal tissues, while the expression levels of other lncRNAs showed no difference and therefore were eliminated in the subsequent study (Figure 1). Next, we detected the four lncRNAs expression in 20 HCC and 20 control plasma. Amongst them, *TUG1* and *linc-ROR* were not detectable in plasma, *ZFAS1* was significantly up-regulated in plasma of tumor patients, while *GAS5* showed no significant changes (Figure 2). Thus, we chose *ZFAS1* for our further analysis.

### Analysis of stability of *ZFAS1* in whole blood and plasma

**Figure 3:**
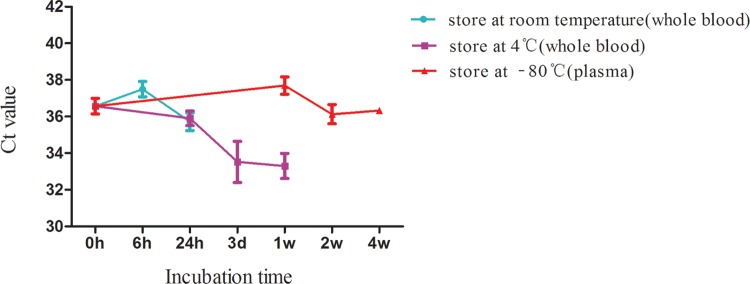
The effect of storage conditions on the expression level of plasma *ZFAS1* The data were analyzed using one-way ANOVA. No significant difference was observed in each group.

Considering that stability is an important pre-requisite for the utility of biomarkers, we analyzed the stability of *ZFAS1* in whole blood and plasma. Blood samples were collected from four healthy humans (three men and one woman) and divided into three groups. Amongst the three groups, two groups were stored in whole blood form at either room temperature or 4°C and one group was stored in plasma form at −80°C. The storage conditions were as follows: room temperature for 0, 6, and 24 h; 4°C for at 0, 24 h, 3 days, and 1 week; −80°C for 0 h, 1, 2, and 4 weeks. The effects of storage conditions on the expression of *ZFAS1* were shown in Figure 3. Results indicated that keeping plasma at −80°C for 1 month had no effect(s) on *ZFAS1* level. Although prolonged incubation of whole blood at room temperature for up to 24 h or at 4°C for 1 week did not change plasma *ZFAS1* levels significantly (*P*>0.05), the plasma level of *ZFAS1* has an increase tendency when they are incubated longer than 24 h in the whole blood form, which reminds us that we should collect and handle the samples within 24 h from drawn blood.

### Correlation between plasma levels of *ZFAS1* and the indexes of peripheral blood cells

**Figure 4:**
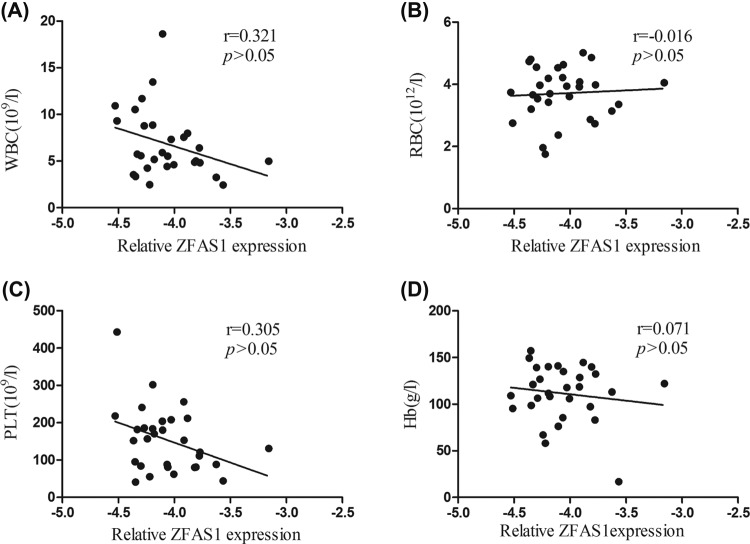
Correlation between plasma *ZFAS1* relative expression and the hematocytes of peripheral blood in HCC patients (**A**) Correlation between plasma *ZFAS1* level and WBC; (**B**) correlation between plasma *ZFAS1* level and RBC; (**C**) correlation between plasma *ZFAS1* level and PLT; (**D**) correlation between plasma *ZFAS1* level and Hb. No significant correlation between plasma *ZFAS1* and any type of peripheral hematocytes was found (*P*>0.05). Correlations were analyzed using the Spearman correlation test.

Previous studies showed that some circulating lncRNAs may be derived from peripheral blood cells [16]. Thus, we evaluated the correlation between the plasma levels of *ZFAS1* and the indexes of peripheral blood cells in 30 HCC patients. No significant correlation between the plasma *ZFAS1* levels and indexes of whole blood were found (*P*>0.05, Figure 4).

### Validation of plasma *ZFAS1* expression level in a large scale

To validate the expression of *ZFAS1* is indeed up-regulated in HCC patients, we measured the levels of plasma *ZFAS1* in 79 control cases, 75 hepatitis B and cirrhosis patients, and 60 HCC patients by RT-qPCR. The main demographic and clinical information of the included subjects were presented in Table 2. No differences existed in gender, age, smoking, and alcoholism whereas a significant difference in alanine aminotransferase (ALT), aspartate aminotransferase (AST), TBIL, γ-glutamyl transferase (GGT), and glucose (GLU) amongst the groups were found.

**Table 2:** Characteristics of the studied participants

Characteristics	HCC	Hepatitis B and cirrhosis	Control	*P*
	*n*=60	*n*=75	*n*=79	
Gender				0.569^1^
Male	50	59	60	
Female	10	16	19	
Age				0.810^1^
<55	29	35	34	
≧55	31	40	45	
Smoking				0.516^1^
Negative	33	40	49	
Positive	27	35	30	
Alcoholism				0.604^1^
Negative	41	50	48	
Positive	19	25	31	
ALT (U/l)	50 (28, 78)^4^	24 (20,38)^3^	20(16,26)	<0.01^2^
AST (U/l)	52 (34, 98)^4^	38 (25,48)^4^	21(20,25)	<0.01^2^
TBIL (μmol/l)	20 (14, 26)^3^	24 (15,34)^4^	12 (10,15)	<0.05^2^
GGT (U/l)	150 (68, 258)^4^	38 (20,128)^3^	21(19,37)	<0.01^2^
GLU (mmol/l)	5.2 (4.5, 6.1)^3^	4.8 (4.0,6.0)	4.4 (4.2,5.3)	<0.05^2^

Data are presented as median (25 percentiles, 75 percentiles). ^1^Chi-square test. ^2^Kruskal–Wallis. ^3^*P*<0.05. ^4^*P*<0.01 compared with Control.

**Figure 5:**
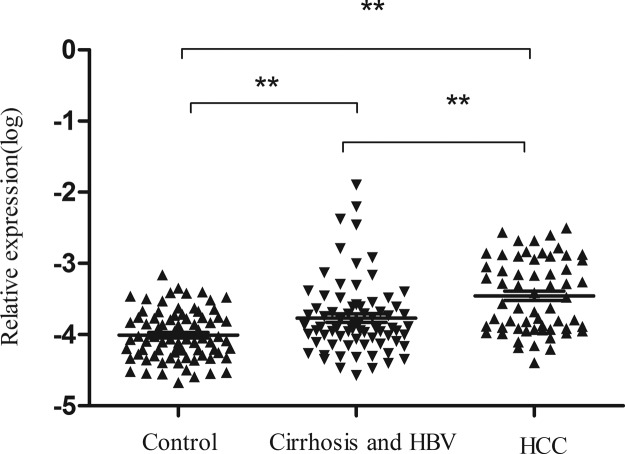
*ZFAS1* expression levels in plasma amongst subgroups *ZFAS1* expressions in HCC patients were higher than that in hepatitis B and cirrhosis and the control groups. The data were analyzed using one-way ANOVA. **P<0.01.

As shown in Figure 5, plasma *ZFAS1* in HCC is higher than that in control group (*P*<0.001) or in hepatitis B and cirrhosis groups (*P*<0.001). Expression of plasma *ZFAS1* in hepatitis B and cirrhosis group was also higher than that in the healthy controls (*P*<0.001). The association between plasma *ZFAS1* expression and clinical characteristics in HCC patients is summarized in Table 3. Results revealed that plasma level of *ZFAS1* was correlated to AFP, whereas no other relevance was found between *ZFAS1* level and other clinical parameters.

**Table 3:** Association between plasma *ZFAS1* expression and clinical characteristics in HCC patients

Characteristics	*n*	*ZFAS1* relative expression(log)		
		Mean ± S.D.	*t*	*P*
Plasma**			−7.60	<0.001
HCC	60	−3.45 ± 0.51		
Healthy controls	79	−4.01 ± 0.35		
Gender			−0.735	0.465
Male	54	−3.47 ± 0.51		
Female	6	−3.30 ± 0.55		
Age			−0.942	0.35
<55	17	−3.55 ± 0.56		
≧55	43	−3.41 ± 0.49		
HBV DNA (IU/ml)			−0.704	0.485
<500	24	−3.49 ± 0.50		
≧500	23	−3.40 ± 0.45		
ALT (U/l)			−0.044	0.965
<46	29	−3.46 ± 0.53		
≧46	31	−3.45 ± 0.50		
AST (U/l)			0.488	0.628
<46	25	−3.42 ± 0.46		
≧46	35	−3.48 ± 0.55		
GGT (U/l)			−0.53	0.598
<55	20	−3.50 ± 0.48		
≧55	40	−3.43 ± 0.53		
AFP (ng/l)*			−2.91	0.006
<200	20	−3.64 ± 0.36		
≧200	26	−3.26 ± 0.49		
CEA			1.086	0.283
<5	42	−3.41 ± 0.53		
≧5	9	−3.62 ± 0.51		

Data are shown as mean ± S.D. Since we failed to collect all characteristics of the HCC patients, the total number may not be 60. Abrreviations: CEA, carcinoembyonic antigen *P<0.05, **P<0.001.

### Diagnostic value of plasma *ZFAS1* for HCC

**Figure 6:**
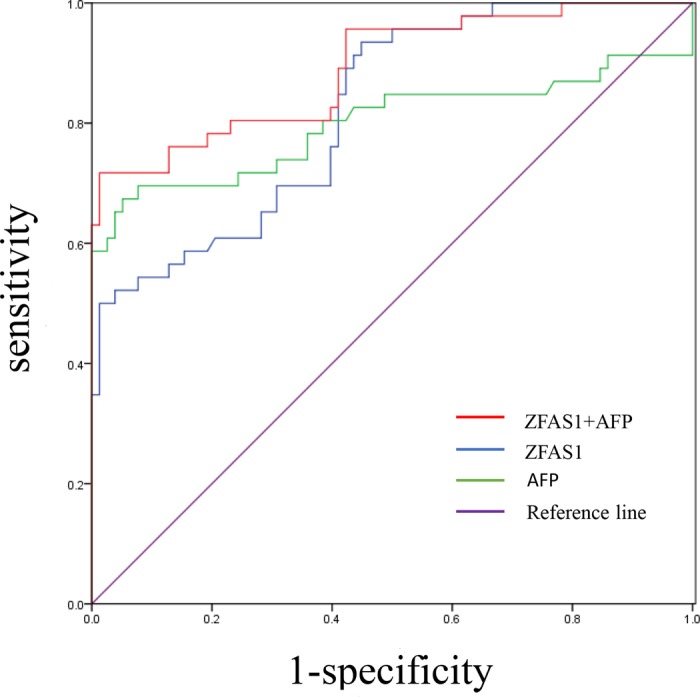
Diagnostic value of plasma *ZFAS1*, serum AFP alone, and combination of these two markers for the detection of HCC The AUC of *ZFAS1* and AFP to distinguish HCC from healthy controls is 0.801 and 0.798, respectively. The combined AUC of these two biomarkers is 0.891. The AUC was got by performing ROC curve analysis.

To assess the diagnostic value of plasma *ZFAS1* for HCC patients, ROC curves were constructed. The area under curve (AUC) of *ZFAS1* to distinguish HCC from healthy controls was 0.801 (95% CI: 0.724–0.875) at the cut-off value of −4.006 with the optical sensitivity and specificity of 55.7 and 90.0%, respectively. AFP is the most commonly used clinical index to screen HCC, so we also assessed the diagnostic value of AFP in HCC. The AUC of AFP was 0.798 (95% CI: 0.700–0.897). In our study, an association between *ZFAS1* and AFP was found, so we combined the two indexes to diagnose HCC. The AUC was high to 0.891 (95% CI: 0.829–0.953), which indicated that the combination of *ZFAS1* and AFP have a stronger diagnostic efficiency than *ZFAS1* or AFP alone (Figure 6). In conclusion, our data validated that *ZFAS1* may serve as a novel diagnostic biomarker for HCC.

## Discussion

In the last few decades, a large number of researches have been performed to find suitable biomarkers for the diagnosis of HCC [25], however, the results are far from satisfactory. Clinically, AFP is the most frequently used liquid biomarker to diagnose HCC, but the sensitivity is low [26]. LncRNAs are a type of RNA molecule longer than 200 nts in length with no protein-coding ability [27]. A handful of studies have showed that lncRNAs played vital roles in many biological processes, especially in tumorigenesis. Circulating biomarkers including lncRNAs are one of the most promising markers of diagnosis since it is easy, convenient, and minimally invasive. Several circulating lncRNAs have already been identified as potential tumor markers for cancers [28,29]. However, studies about circulating lncRNAs in diagnosis of HCC patients are few. In the present study, we aimed to explore favorable circulating lncRNAs for the diagnosis of HCC.

In the present study, six cancer-related lncRNAs were selected to explore their expression profiling in HCC tissues. Two lncRNAs were discarded due to no significant difference in HCC tissues and paired adjacent tissues. Then the remaining four lncRNAs were further measured in plasma from 20 HCC patients and 20 healthy subjects. Unfortunately, *TUG1* and *linc-ROR* were not detectable in plasma and *GAS5* was not differently expressed between HCC patients and healthy controls. Thus, *ZFAS1* was finally identified. We further explored the expression profiling of *ZFAS1* in 79 control cases, 75 hepatitis B and cirrhosis patients, and 60 HCC patients, the expression of *ZFAS1* is up-regulated from healthy, liver disease, and HCC step by step, which further validated our previous results. A study conducted by Li et al. [19] revealed that *ZFAS1* is up-regulated in HCC tissues and up-regulation of *ZFAS1* is correlated with poor prognosis in HCC. Our experiment is not only a validation of their study but also an extension of theirs by exploring the diagnostic role of *ZFAS1* in HCC.

To assess the diagnostic value of *ZFAS1*, ROC analysis was conducted. Results showed that AUC was 0.801 (95% CI: 0.724–0.875), suggesting that plasma *ZFAS1* could be a promising tumor marker for HCC detection. In addition, the AUC was 0.891 (95% CI: 0.829–0.953) when we use of *ZFAS1* and AFP in combination. Therefore, combing the two markers together can apparently improve the diagnostic power.

The data from our study showed that *ZFAS1* was highly expressed in plasma of HCC patients, while *TUG1* and *linc-ROR* were not detectable. Interestingly, Ma et al. [30] found that lncRNA *TUG1* in plasma was correlated with disease status in osteosarcoma. Isin et al. [31] demonstrated that the expression level of *TUG1* was up-regulated in the plasma of multiple myeloma patients compared with the healthy controls. A recent study performed by Schlosser et al. [32] showed that despite pre-amplification, the expression of *TUG1* and *linc-ROR* in plasma were surprisingly sporadically detectable, which is consistent with our study. It is well known that hemolysis and cellular/platelet contamination of plasma can lead to artificially elevated measurements of lncRNAs. Thus the speed and duration of centrifugation used to prepare the plasma may influence the results. Therefore, the different results between our findings and prior studies might be due to the different preparation methods and quality of plasma. In our study, the plasma was centrifuged twice at 12000 ***g*** for 10 min at 4°C to remove cells, platelets, and cell debris to the maximum extent. Besides, we collected the plasma samples within 12 h. However, the preparation of plasma specimens and collection time used in several prior studies have not been clearly elaborated.

Taken together, we clearly demonstrated that plasma levels of *ZFAS1* are higher in HCC patients than that in healthy controls and in patients with cirrhosis and hepatitis B, and the expression of *ZFAS1* is correlated to serum AFP. In addition, *ZFAS1* could be a good diagnostic marker to distinguish HCC patients from healthy controls. Nevertheless, there is still a long way to go before these findings can be used for clinic. We will prospectively confirm the usefulness of plasma *ZFAS1* in a large number of patients and report on these results in the near future.

## Supporting information

**Supplemental Table 1:** Demographic and clinical characteristics of HCC patients.

## Abbreviations

AFP: α-fetoprotein
AUC: area under curve
HBV: hepatitis B virus
HCC: hepatocellular carcinoma
lncRNA: long non-coding RNA
ROC: receiver operating characteristic

## References

[1] Cadier B., Bulsei J., Nahon P. (2017) Early detection and curative treatment of hepatocellular carcinoma: a cost-effectiveness analysis in France and in the United States. Hepatology 65, 1237–1248 10.1002/hep.28961 28176349

[2] El-Serag H.B. and Rudolph K.L. (2007) Hepatocellular carcinoma: epidemiology and molecular carcinogenesis. Gastroenterology 132, 2557–2576 10.1053/j.gastro.2007.04.061 17570226

[3] Shimagaki T., Yoshizumi T., Harimoto N. (2017) MicroRNA-125b expression and intrahepatic metastasis are predictors for early recurrence after hepatocellular carcinoma resection. Hepatol. Res. 48, 313–321 28984009 10.1111/hepr.12990

[4] Yu J., Han J., Zhang J. (2016) The long noncoding RNAs PVT1 and uc002mbe.2 in sera provide a new supplementary method for hepatocellular carcinoma diagnosis. Medicine (Baltimore) 95, e4436 10.1097/MD.0000000000004436 27495068 PMC4979822

[5] Farvardin S., Patel J., Khambaty M. (2017) Patient-reported barriers are associated with lower hepatocellular carcinoma surveillance rates in patients with cirrhosis. Hepatol. 65, 875–884 10.1002/hep.28770 27531684 PMC5568252

[6] Quagliata L., Matter M.S., Piscuoglio S. (2014) Long noncoding RNA HOTTIP/HOXA13 expression is associated with disease progression and predicts outcome in hepatocellular carcinoma patients. Hepatology 59, 911–923 10.1002/hep.26740 24114970 PMC3943759

[7] Li J., Wang X., Tang J. (2015) HULC and Linc00152 act as novel biomarkers in predicting diagnosis of hepatocellular carcinoma. Cell. Physiol. Biochem. 37, 687–696 10.1159/000430387 26356260

[8] Cao S.W., Huang J.L., Chen J. (2017) Long non-coding RNA UBE2CP3 promotes tumor metastasis by inducing epithelial-mesenchymal transition in hepatocellular carcinoma. Oncotarget 39, 65370–65385 10.18632/oncotarget.18524 PMC5630337 29029437

[9] Quinn J.J. and Chang H.Y. (2016) Unique features of long non-coding RNA biogenesis and function. Nat. Rev. Genet. 17, 47–62 10.1038/nrg.2015.10 26666209

[10] Huarte M. (2015) The emerging role of lncRNAs in cancer. Nat. Med. 21, 1253–1261 10.1038/nm.3981 26540387

[11] Xu J.H., Chang W.H., Fu H.W. (2017) Upregulated long non-coding RNA LOC90784 promotes cell proliferation and invasion and is associated with poor clinical features in HCC. Biochem. Biophys. Res. Commun., 10.1016/j.bbrc.2017.06.141 28651931

[12] Ma X., Wang X., Yang C. (2016) DANCR acts as a diagnostic biomarker and promotes tumor growth and metastasis in hepatocellular carcinoma. Anticancer Res. 36, 6389–6398 10.21873/anticanres.11236 27919960

[13] Komatsu S., Ichikawa D., Takeshita H. (2014) Circulating miR-18a: a sensitive cancer screening biomarker in human cancer. In Vivo 28, 293–297 24815829

[14] Wan L., Kong J., Tang J. (2016) HOTAIRM1 as a potential biomarker for diagnosis of colorectal cancer functions the role in the tumour suppressor. J. Cell. Mol. Med. 20, 2036–2044 10.1111/jcmm.12892 27307307 PMC5082402

[15] Liang W., Lv T., Shi X. (2016) Circulating long noncoding RNA GAS5 is a novel biomarker for the diagnosis of nonsmall cell lung cancer. Medicine (Baltimore) 95, e4608 10.1097/MD.0000000000004608 27631209 PMC5402552

[16] Zhou X., Yin C., Dang Y. (2015) Identification of the long non-coding RNA H19 in plasma as a novel biomarker for diagnosis of gastric cancer. Sci. Rep. 5, 11516 10.1038/srep11516 26096073 PMC4476094

[17] Tong Y.S., Wang X.W., Zhou X.L. (2015) Identification of the long non-coding RNA POU3F3 in plasma as a novel biomarker for diagnosis of esophageal squamous cell carcinoma. Mol. Cancer 14, 3 10.1186/1476-4598-14-3 25608466 PMC4631113

[18] Han L., Ma P., Liu S.M. (2016) Circulating long noncoding RNA GAS5 as a potential biomarker in breast cancer for assessing the surgical effects. Tumour Biol. 37, 6847–6854 10.1007/s13277-015-4568-7 26662314

[19] Li T., Xie J., Shen C. (2015) Amplification of long noncoding RNA ZFAS1 promotes metastasis in hepatocellular carcinoma. Cancer Res. 75, 3181–3191 10.1158/0008-5472.CAN-14-3721 26069248

[20] Yang L., Qiu M., Xu Y. (2016) Upregulation of long non-coding RNA PRNCR1 in colorectal cancer promotes cell proliferation and cell cycle progression. Oncol. Rep. 35, 318–324 10.3892/or.2015.4364 26530130

[21] Tu Z.Q., Li R.J., Mei J.Z. (2014) Down-regulation of long non-coding RNA GAS5 is associated with the prognosis of hepatocellular carcinoma. Int. J. Clin. Exp. Pathol. 7, 4303–4309 25120813 PMC4129048

[22] Huang M.D., Chen W.M., Qi F.Z. (2015) Long non-coding RNA TUG1 is up-regulated in hepatocellular carcinoma and promotes cell growth and apoptosis by epigenetically silencing of KLF2. Mol. Cancer 14, 165 10.1186/s12943-015-0431-0 26336870 PMC4558931

[23] Takahashi K., Yan I.K., Kogure T. (2014) Extracellular vesicle-mediated transfer of long non-coding RNA ROR modulates chemosensitivity in human hepatocellular cancer. FEBS Open Biol. 4, 458–467 10.1016/j.fob.2014.04.007 24918061 PMC4050189

[24] Czarny M.J., Babcock K., Baus R.M. (2007) Hepatocellular carcinomas of the albumin SV40 T-antigen transgenic rat display fetal-like re-expression of lgf2 and deregulation of H19. Mol. Carcinog. 46, 747–757 10.1002/mc.20286 17393425

[25] Li B., Li B., Guo T. (2017) Artificial neural network models for early diagnosis of hepatocellular carcinoma using serum levels of alpha-fetoprotein, alpha-fetoprotein-L3, des-gamma-carboxy prothrombin, and golgi protein 73. Oncotarget, 8, 80521–80530 29113322 10.18632/oncotarget.19298 PMC5655217

[26] Yamashita T., Kitao A., Matsui O. (2014) Gd-EOB-DTPA-enhanced magnetic resonance imaging and alpha-fetoprotein predict prognosis of early-stage hepatocellular carcinoma. Hepatology 60, 1674–1685 10.1002/hep.27093 24700365 PMC4142120

[27] Audas T.E. and Lee S. (2016) Stressing out over long noncoding RNA. Biochim. Biophys. Acta 1859, 184–191 10.1016/j.bbagrm.2015.06.010 26142536 PMC9479161

[28] Deng H., Wang J.M., Li M. (2017) Long non-coding RNAs: new biomarkers for prognosis and diagnosis of colon cancer. Tumour Biol. 39, 1010428317706332 10.1177/1010428317706332 28643604

[29] Hu H.B., Jie H.Y. and Zheng X.X. (2016) Three circulating LncRNA predict early progress of esophageal squamous cell carcinoma. Cell. Physiol. Biochem. 40, 117–125 10.1159/000452529 27855375

[30] Ma B., Li M., Zhang L. (2016) Upregulation of long non-coding RNA TUG1 correlates with poor prognosis and disease status in osteosarcoma. Tumour Biol. 37, 4445–4455 10.1007/s13277-015-4301-6 26499949

[31] Isin M., Ozgur E., Cetin G. (2014) Investigation of circulating lncRNAs in B-cell neoplasms. Clin. Chim. Acta 431, 255–259 10.1016/j.cca.2014.02.010 24583225

[32] Schlosser K., Hanson J., Villeneuve P.J. (2016) Assessment of circulating LncRNAs under physiologic and pathologic conditions in humans reveals potential limitations as biomarkers. Sci. Rep. 6, 36596 10.1038/srep36596 27857151 PMC5114641

