# Supplementary material for: Identification of long non-coding RNA ZFAS1 as a novel biomarker for diagnosis of HCC
Source: Biosci Rep. 2018 Jul 18;38(4):BSR20171359. doi: 10.1042/BSR20171359 (PMC6050191; doi:10.1042/BSR20171359)
Supplement: Supplementary file 1 [file bsr20171359_Supp1.pdf]

Supplemental Table 1. Demographic and clinical characteristics of HCC patients.

NO.	Sex	Age	Smoking	Alcoholism	Differentiation	TNM	Size(cm)	ALT(U/l)	AST(U/l)	TP(g/l)	ALB(g/l)
1	Male	43	No	No	poorly	III	13*10	24	25	63.6	41.2
2	Male	57	Yes	Yes	moderate	II	8*7*5	50	65	65.3	34.5
3	Male	52	Yes	No	moderate	II	9.6*8	80	57	69.3	39.3
4	Male	52	No	No	moderate	III	9.7*16.3*13.3	79	108	638	32
5	Male	61	Yes	Yes	moderate	III	15.8*11.2	148	100	58.6	39
6	Female	63	Yes	No	poorly	II	13*12	57	57	57.5	35.3
7	Male	57	Yes	No	moderate	II	3.2*4.3	49	43	69.4	27.5
8	Male	79	No	Yes	moderate	III	13.6*9.6	21	24	58	32.5
9	Male	64	Yes	Yes	moderate	II	7*6*5	68	67	72.5	42.5
10	Male	70	Yes	No	moderate	III	16.3*13.6	50	66	79.2	40
11	Female	51	Yes	No	moderate	III	12.2*11.4	24	92	57.8	36.6
12	Male	51	No	No	moderate	II	3.5*2.1	48	32	69.1	41
13	Male	55	Yes	Yes	moderate	II	10*9*8	41	32	73.2	47.4
14	Male	57	Yes	Yes	moderate	III	19.5*15.1	38	76	64.6	39.9
15	Male	42	No	Yes	poorly	III	13*10	54	117	68.4	36.7
16	Male	53	No	No	moderate	II	9.0*8.0	60	42	60.1	38.2
17	Male	86	No	No	moderate	I	10*12	16	27	80.2	33.8
18	Male	60	No	Yes	well	I	4*3	68	51	74	47.8
19	Male	55	No	No	poorly	III	1.4*2.5	39	29	66.6	42.4
20	Male	75	No	No	moderate	III	7.5*5.8	26	24	64.7	36.6
21	Male	47	Yes	Yes	moderate	II	10*8	33	55	65.3	36
22	Male	47	No	No	poorly	II	3.8*3.1；4*2	59	87	42.3	25.1
